# A review of methods to estimate the visibility factor for bias correction in network scale-up studies

**DOI:** 10.4178/epih.e2018041

**Published:** 2018-08-16

**Authors:** Aliakbar Haghdoost, Milad Ahmadi Gohari, Ali Mirzazadeh, Farzaneh Zolala, Mohammad Reza Baneshi

**Affiliations:** 1 Modeling in Health Research Center, Institute for Futures Studies in Health, Kerman University of Medical Sciences, Kerman, Iran; 2 HIV/STI Surveillance Research Center, WHO Collaborating Center for HIV Surveillance, Institute for Futures Studies in Health, Kerman University of Medical Sciences, Kerman, Iran; 3 Department of Epidemiology and Biostatistics and Institute for Global Health Sciences, University of California, San Francisco, CA, USA; 4 Social Determinants of Health Research Center, Institute for Futures Studies in Health, Kerman University of Medical Sciences, Kerman, Iran

**Keywords:** Network scale-up, Visibility, Transparency, Systematic review, Bias

## Abstract

Network scale-up is an indirect size estimation method, in which participants are questioned on sensitive behaviors of their social network members. Therefore, the visibility of the behavior affects the replies and estimates. Many attempts to estimate visibility have been made. The aims of this study were to review the main methods used to address visibility and to provide a summary of reported visibility factors (VFs) across populations. We systematically searched relevant databases and Google. In total, 15 studies and reports that calculated VFs were found. VF calculation studies have been applied in 9 countries, mostly in East Asia and Eastern Europe. The methods applied were expert opinion, comparison of NSU with another method, the game of contacts, social respect, and the coming-out rate. The VF has been calculated for heavy drug users, people who inject drugs (PWID), female sex workers (FSWs) and their clients, male who have sex with male (MSM), alcohol and methamphetamine users, and those who have experienced extra-/pre-marital sex and abortion. The VF varied from 1.4% in Japan to 52.0% in China for MSM; from 34.0% in Ukraine to 111.0% in China for FSWs; and from 12.0% among Iranian students to 57.0% in Ukraine for PWID. Our review revealed that VF estimates were heterogeneous, and were not available for most settings, in particular the Middle East and North Africa region, except Iran. More concrete methodologies to estimate the VF are required.

## INTRODUCTION

Population size estimation (PSE) is a crucial step in creating knowledge about various groups. This is very important for advocacy, planning, and resource allocation [1]. There are several direct methods of carrying out PSE. The major direct methods include census and enumeration. Direct methods require access to and successful recruitment strategies of members of the populations of interest [2]. To obtain precise PSE results from direct methods, a large sample size is needed, which is also challenging. Conducting household surveys of the general population and directly asking about participants’ sensitive personal behaviors (such as drug injection or paid sex) may also result in under-reporting due to social desirability bias and the stigmas surrounding such behaviors [3]. In many countries, including those with Islamic culture and social norms, direct questions about such stigmatized behaviors are especially prone to bias [4].

Given the limitations of direct methods of PSE, several indirect methods have been developed, such as the capture-recapture, multiplier, and network scale-up (NSU) methods. The application of the first 2 methods depends on the availability of registry systems, and the ability to conduct a survey among members of the population of interest. These requirements restrict the practicality of these methods in many countries.

In contrast, in NSU, a random sample of the general population is asked to report the number of individuals in their active social network who are members of the population of interest. This method assumes that the size of the population of interest reported in the networks reflects the size of the population of interest in the community [5].

The NSU method has several benefits in comparison to other methods. Importantly, it can be carried out using a sample of the general population. With no additional costs, it can be used to estimate the size of several populations of interest. It has been implemented successfully in many countries and for several populations of interest. Examples of previous NSU studies include studies in the US to estimate hidden subgroups, including human immunodeficiency virus (HIV)-positive individuals [2], heroin or crack users [6], and female who have been raped [7]; studies in Moldova [8], Ukraine [9], Thailand [10], and Rwanda [11] to estimate the population size of injection drug users, female sex workers (FSWs), and male who have sex with other male (MSM); a study in Brazil [12] conducted to estimate the population size of heavy drug users; a study in Japan [13] to estimate the population of MSM; a study in Georgia [14] to estimate the number of people who inject drugs (PWID) and MSM, and studies in Iran to estimate the number of PWID [15], FSWs [16], alcohol users [17] and female who have undergone an abortion [18].

A key assumption in an NSU study is that participants are aware of the sensitive behaviors of people in their network. This is a difficult assumption to make, since it is very likely that sensitive information is not always transmitted among members of a network [12]. This issue is referred to as the transparency barrier bias. This bias results in an underestimation of the size of populations of interest. To correct for this bias, the crude NSU estimates are divided by a correction factor called the visibility factor (VF). In brief, VF is the proportion of people in the networks of members of a population of interest who are aware of their behavior [19]. For example, a visibility of 50% means that only 50% of people in the networks of PWID know that the person in question is injecting drugs. This indicates that the population size estiamtion of NSU needs to be divided by 0.5, or doubled, to account for this bias.

So far, methods to estimate the VF correction factor have not been assessed and presented systematically. The aim of the current review is to briefly review the methods used to estimate VF, to provide a summary of VF reports, and to assess the heterogeneous factors that cause VF to vary across settings and populations.

## MATERIALS AND METHODS

### Searches and study selection

We searched PubMed, Scopus, Embase, and Web of Science (WoS) databases in November 2017. We did not limit the publication time. In the first step, we used the MeSH database (https://www.ncbi.nlm.nih.gov/mesh/) and searched for equivalents of “network scale-up,” “visibility,” “transparency,” and “social respect.” The keywords suggested by MeSH were not relevant to PSE studies. For example, for visibility, the terms “lightening” and “klean-prep” were suggested.

The language that we used in our search was English. First, we searched for “network scale-up.” In total, 37 manuscripts were found in PubMed. Two of them were in Chinese with an English-language abstract. The corresponding searches in WoS, Embase, and Scopus led to 30, 32, and 71 results, respectively. Omitting duplicates, a total of 76 manuscripts were found.

The key terms we used in our search and the corresponding number of manuscripts found were: “network scale-up” AND “visibility” AND “transparency,” which led to no manuscripts; “network scale-up” AND “visibility,” which in total provided 5 manuscripts; “network scale-up” AND “transparency,” which provided 2 manuscripts in Scopus and 1 in other databases; “network scale-up” AND “social respect,” which found 1 manuscript in all 4 databases, and “network scale-up” AND “size estimation,” which gave 21 manuscripts in PubMed, 16 in WoS, 23 in Scopus, and 18 in Embase. We also examined the citations, but no further relevant manuscripts were found.

To expand our search strategy, we also searched the Google search engine using the above key terms. We found 4 reports that applied some correction factors and were available as free PDF documents. Altogether, 80 unique manuscripts and reports were found.

### Data extraction

MA and MRB, the coauthors on this paper, independently read the full reports and full text of all papers. Only 15 documents reported a VF, and were therefore selected and included in our analysis. We extracted information such as the method used to estimate the VF and the hidden groups that were of interest.

## RESULTS

Different methodologies have been applied to estimate the VF. In some of these methods, direct contact with members of a hidden population is required. The game of contacts [12] and social respect methods [20] fall into this category. Other methods do not involve contact with members of the hidden group. These methods include expert opinion [18] and comparison of the results of 2 size estimation methods [21]. A summary of these methods, with their advantages and disadvantages, is provided in Table 1.

We found 2 manuscripts in Chinese with an English abstract. One of them estimated the number of FSWs and their clients in the city of Taizhou [22]. Based on the information in the abstract, we concluded that no correction factor was calculated. In the other Chinese-language study, the size of the MSM population in Chongqing Province was estimated [23]. The authors briefly stated that the size was adjusted using the respective levels but they did not explain how it was calculated.

We found 15 studies that estimated the VF or adopted it from other studies to correct their size estimates (Table 2). The hidden groups that were of interest were PWID [8-10,15,24,25], FSWs [11,16,19,26], MSM [9,11,13,14,20,26], heavy drug users [12,24,26], alcohol users [17], and those who have had an abortion [18,27]. An innovative application was found in which an NSU study using VF was conducted to assess the completeness of a cancer registry [28]. These studies were conducted in 8 countries. Below, the methodologies of these studies and their main findings are briefly reviewed.

The results are organized by the method of estimating the VF. In Table 2, findings are provided by risky behavior. We should mention that various terms for this concept, such as the VF, correction factor, and social respect factor, are used in the literature. We translated all reported values to VF, so that the crude estimate divided by the VF provided the adjusted size. For example, a correction factor or social respect factor of 2 was translated into a VF of 50%.

### Expert opinion

In Middle East societies with Islamic culture, access to these groups can be extremely difficult. An ad hoc method is to approach field experts and ask their opinion about the visibility of the behavior of interest.

#### Iran

A national study was conducted in Iran to estimate the annual incidence of abortion [18]. The authors approached 34 midwives and gynecologists, explained the concept of transparency, and asked those 34 experts about the minimum and maximum visibility of abortion in Iran’s culture. The average of their responses was applied as the VF. Therapeutic abortion was defined as abortion with medical indication (AWMI+). Spontaneous and intentional abortions were considered as abortion without medical indication (AWMI-). The minimum and maximum VFs for AWMI+ were 43.0 and 75.0%. The corresponding values for AWMI- were 34.0 and 20.0%.

### Comparison of the results of network scale-up with another size estimation method

Some authors have compared estimates derived using NSU with another method and used the ratio of the estimates as a measure of visibility. NSU has been compared with the cross-wise (CW) method [21], the proxy respondent method (PRM) [25], and the multiplier method [8]. These authors assumed that other methods were less prone to transparency bias. To justify their assumptions, we briefly explain how these methods work.

#### Iran

The basis of the CW method is to match a sensitive question with a non-sensitive question. The non-sensitive question must be independent of the sensitive item. A random sample of respondents is asked to answer both questions simultaneously. The respondent is asked to choose the option “A” if his/her answers to both questions are the same (both yes or both no) and to choose “B” if his/her answers are different (one of them yes, another is no). According to the frequency of option A, the prevalence of the sensitive trait is estimated. The ratio of the prevalence obtained using NSU to that obtained using the CW method has been reported as a measure of VF.

In 2012, we recruited 563 university students and estimated the scale of different risky behaviors using the CW and NSU methods. The prevalence of alcohol consumption, amphetamine use, and extra-/pre-marital sex in the previous year (even 1-episode) were estimated [21]. Data were collected using an anonymous researcher-developed questionnaire. The annual prevalence of alcohol consumption was estimated as 16.8% using CW and 8.1% using NSU, giving a VF of 48.0%. The corresponding figures for methamphetamine use were 7.2 and 1.2%, resulting to a VF of 17.0%. The annual prevalence of extra-/pre-marital sex was estimated as 12.4 and 7.1%, respectively, suggesting a VF of 57.0%.

#### Iran

The PRM is a modified version of the NSU method in which there is no need to estimate the size of individuals’ social network. In this method, randomly-sampled respondents (proxy respondents) are asked about whether they have a close friend with a specific name and whether that person practices the risky behavior of interest. The main assumption of the PRM method is that the random sample of the selected respondents and alters form a representative sample of the community [25]. The ratio of estimates made using NSU to those made using PRM has been reported as a measure of the VF.

Sheikhzadeh et al. [25] recruited 500 university students and compared the results of NSU with those obtained using the PRM. The replies of 420 students with complete data were analyzed. In the NSU analysis, participants were asked about the count of their close friends and the number of them who engaged in risky behaviors (even 1-episode in the last year). In the PRM exercise, 30 male and 30 female names were written on 12 cards. Male students were asked to select one of the male cards, and to randomly select one of the names written on it. The research team asked the respondent to select one of his close university friends with that name. Next, the respondent was asked whether the selected person engaged in the risky behaviors of interest. The same process was applied for female students. The VF of alcohol consumption for female and male students was estimated as 19.0 and 48.0%, respectively. The corresponding figures for extra-/pre-marital sex were 27.0 and 56.0%, respectively.

#### Moldova

The multiplier method depends on comparing the information derived from 2 distinct and independent data sources that overlap in a known way: a list followed by a survey. A list of individuals in the target population who have either accessed some type of services or received a unique object is the first source of data. The second source of data is a representative survey of the target population. Multiplying the number of those who received the service or object by the inverse of the proportion reporting that they received the service or object yields the population size [16].

In Moldova in 2009, 1,969 persons aged 15 to 64 were recruited [8]. Multiple distinct methods, including the capture-recapture, multiplier, and NSU methods, were applied. In the report from Moldova, no information about the calculation of the correction factor by means of comparing 2 methods was provided. Another report that shared experiences in PSE, published by Joint United Nations Programme on HIV/AIDS (UNAIDS), mentioned that the results from NSU underestimated the population size compared to estimates derived using the multiplier method. The UNAIDS claimed that authors calculated the ratio of the multiplier estimate to the NSU estimate as an undercount coefficient. This correction factor was applied to the remaining regions in which size estimation had not been conducted using the multiplier method. This approach enabled sub-national and national estimates.

### Game of contacts

The main method used to calculate the visibility is referred to as the “game of contacts.” To measuremeasure the VF, a list of names is selected. Members of the hidden group, known as egos, are asked about the total number of their acquaintances with any of the selected names (reference group), and of those, how many are aware that the ego is a member of the hidden sub-population. For each name (shown by *j*), the replies of all subjects are aggregated. The VF is calculated by dividing the total number of aware alters (a_j_) by the total number of alters (t_j_) (where *j* stands for the name):

$$VF_{j}=\frac{\sum_{j}a_{j}}{\sum_{j}t_{j}}$$

The mean of *VF_j_* is used as the final VF.

#### Brazil (Curitiba)

In a study conducted in Brazil, heavy drug users were defined as individuals who had injected drugs at least once in the last 6 months and/or had used illicit drugs other than marijuana on at least 25 days during the last 6 months. A respondent-driven sampling method was used to recruit 294 samples. The overall VF was estimated at 76.0%. Cocaine paste and marijuana use had the lowest and highest VFs, at 72.0 and 78.0%, respectively [24].

#### Iran

In Iran, to measure the VF for PWID and FSWs, 163 PWIDs and 76 FSWs who received services from drop-in clinics (DICs) were recruited [19]. Using a structured face-to-face interview, data were collected by a trained same-sex interviewer. Authors selected 10 one-part male and 10 one-part female names with a frequency of 0.1 to 4.0% in the community to maximize the precision. To estimate the 95% uncertainty intervals (UIs), a bootstrap procedure was applied. The VFs for PWID and FSWs were 54.0% (95% UI, 50.0 to 58.0) and 45.0% (95% UI, 42.0 to 48.0), respectively. These correction factors were used to adjust the results of a Iranian national study [30].

Although experts’ opinion should guide the study of this issue, they might overestimate the visibility, as experts are in touch with the hidden group. Approaching private and public health centers, Zamanian et al. [27] recruited 222 Iranian female who had an abortion within the last year. The sample size for each type of abortion was about 75. The VFs for intentional, therapeutic, and spontaneous abortion were 8.0, 60.0, and 50.0%, respectively.

#### Georgia

In a household survey in Tbilisi, 1,015 adults were recruited to estimate the population size of PWID. In an independent survey of 210 MSM, the VF was estimated as 26.0% [14].

In another study, the game of contacts was applied among 1,951 PWIDs recruited from 7 cities in Georgia. The results varied from 32.0 to 46.0%. For the aggregated data, the VF was estimated as 40.5% (95% UI, 38.3 to 42.6) [29].

### Social respect

Another method to estimate transparency is referred to as social respect. Respondents are asked about their attitude towards these hidden groups, on a scale with values of 1 (very low), 2 (low), 3 (medium), 4 (high), and 5 (very high). Then the number of MSM that a participant knows is weighted by his or her respect level (shown by *W_i_*). This is defined as the average number of MSM known to participants with a given respect level divided by that of the respondents with medium level [9]:

$$\mathrm{That}\;\mathrm{is},\;W_{i}=\frac{M_{i}}{M_{3}}$$

For example, if the average number of FSWs known to the respondents with respect levels of *i* and 3 is 0.4 and 0.8, respectively, then replies from the former group were weighted using a factor of 0.4/0.8=0.5. The weighted replies are finally used to calculate the size of the hidden group.

#### China (Shanghai)

Nearly 4,000 respondents were recruited to estimate the number of MSM in Shanghai [20]. Among them, 622 admitted that they knew MSM. The crude amount of MSM was calculated as 18,881. From the replies of those 622 participants, the authors applied a social respect factor of 52.0% to adjust the estimates. This nearly doubled the size (36,354 vs. 18,881). This corresponded to 0.3% of the male adult population of Shanghai.

#### China (Chongqing)

A multistage random sampling method among 2,957 members of the general population aged between 18 and 60 years was conducted. In total, 229 participants reported that they knew FSWs. Interestingly, the adjusted estimate was lower than the crude estimate, as the crude estimate was divided by 111.0%. With respect to clients of FSWs, 480 participants reported that they knew clients of FSWs, and the VF was estimated as 45.0%. The VF for drug use was 50.0%. The number of participants who admitted knowing PWID and MSM was 113. The corresponding VFs were 50.0 and 44.0%, respectively.

#### Rwanda

In Rwanda, both the known-reference-group and summation methods were applied to estimate network size [11]. In addition, standard and meal definitions were applied to define ‘know.’ Using the known-reference-group approach with the meal definition, the number of MSMs was estimated at the range of 100-4,700. In a pilot study among 17 MSMs, the VF was calculated as 20.0%. However, this figure was not used, as the sample was not representative of the MSM population. Instead, the authors assumed that the VF ranged from 5.0 to 100.0% and graphically showed the influence of the VF on estimates.

#### Georgia

Sulaberidze et al. [14] applied multiple methods to triangulate the number of MSM in Tbilisi, Georgia. In their NSU exercise, a household survey was applied to in which 1,015 adults were recruited. Following the game of contacts, 210 MSMs were approached, and the VF was calculated as 26.0%.

#### Ukraine

In 2009, a national study was conducted in Ukraine to estimate the size of the PWID, FSW, and MSM populations [9]. Around 1,000 respondents aged above 14 were recruited. The results of this study were corrected using 2 factors: social respect and visibility. Social respect was calculated as explained above, and the crude size estimates were adjusted. To calculate the VF, 28 PWID, 21 FSWs, and 108 MSM were asked to provide a list of their acquaintances. This was followed by asking how many of them were aware of their behavior. The social visibility of PWID, FSWs, and MSM was 57.0, 34.0, and 24.0%, respectively, corresponding to correction factors of 1.7, 2.9, and 4.2. To minimize the impact of using a small and non-representative sample, the results of other surveys among MSM were considered and the above correction factors were slightly modified. The final correction factors were 1.5, 2.0, and 2.3, respectively.

#### Moldova

In Moldova in 2009, 1,969 persons aged 15 to 64 were recruited. Multiple methods, including the capture-recapture, multiplier, and NSU methods, were applied [8]. To assess the impact of transmission error on the estimation of PWID, the social respect correction factor was calculated. As the respect questions were on a scale of 1 to 5, the authors assumed that on average people with respect of 1 for PWIDs reported only 20.0% of the most-at-risk populations in their network. Thus, they developed a second weighting system: V1=0.6, V2=0.8, V3=1, V4=1.2, and V5=1.6. PSE was applied in 8 areas. The social respect adjustment was calculated and applied in each of the 8 regions separately. The lowest and highest social respect values were 5.0 and 389.0%, respectively. This means that the size of the population in one area was multiplied by 20, while that for another was divided by approximately 4. The second adjustment was applied only in a central rural area.

### Coming-out rate

Ezoe et al. [13] proposed an index that they named the “coming-out rate.” In this method, the average number of acquaintances of MSM who are aware of their behavior is divided by the average total number of persons in their network. Clearly, the coming-out rate method is essentially the game of contacts, in which the denominator comes from a general population survey rather than hidden population.

#### Japan

Ezoe et al. [13] conducted an internet survey among 1,500 Internet users. The crude MSM rate among the total male population was estimated as 0.04%. To adjust this, authors used data from a previous study that examined HIV prevention behaviors among MSM. In that Internet survey, based on an analysis of 5,731 valid responses, it was seen that on average, 5.1 people in the network of MSMs were aware of their behavior. The authors divided 5.1 by the average network size (364) and defined it as the ‘coming-out rate.’ This gave a correction factor of 1.4%. Dividing the unadjusted prevalence by this correction factor, the prevalence was calibrated at 2.9%.

## DISCUSSION

While NSU is a promising tool for estimating the size of hidden groups, failure to consider its assumptions results in biased estimates. One of its main assumptions is that respondents are aware of the behavior of their acquaintances. This assumption is difficult to meet, and therefore, an appropriate correction factor should be applied. Our review showed that different methodologies have been applied to address the visibility of hidden characteristics. This makes the comparison of results difficult. The game of contacts was the method most frequently applied.

Some studies adopted correction factors from other studies. For example, in Tabriz, 500 subjects were recruited to estimate the prevalence of several risky groups, including PWID and FSWs [31]. They used the correction factors reported by Maghsoudi et al. [19] to take into account the issue of visibility. Similarly, in Iran’s national FSW study, the correction factor presented by Maghsoudi et al. [19] of 45.0% was applied. In Iran’s national illicit drug use size estimation exercise [15], we adopted correction factors from a study conducted in Brazil and the study of Maghsoudi et al. [19] In a national study of alcohol consumption in Iran, the results of the Brazilian study was applied [17]. As social and cultural issues affect the level of visibility, there is room to design concrete studies to measure the VF in different settings.

Due to lack of access to hidden groups, some authors adopted an innovative method that compares NSU estimates with other methods. It seems reasonable to assume that the CW method should provide a higher estimate than NSU. This is because in CW respondents reveal information about themselves, and in NSU they speak about their network. The same is true for the PRM. We expect higher estimates in the PRM, as subjects reply on behalf of a randomly selected close friend, and therefore it is reasonable to assume that the visibility is higher than obtained using NSU. While both the NSU and PRM methods are prone to transparency bias, we believe that the PRM is less affected than NSU, and therefore the ratio of estimates provides a rough estimate of the VF. We should emphasize that the comparison methods have been applied in limited populations. For example, our research team implemented this method among university students, but not in the general population. Therefore, the derived VFs are applicable to similar populations. We believe that it would also be interesting to investigate the practicality of this method in the general population.

An interesting finding was the VF for FSWs in the study conducted in Chongqing, China, where the crude and adjusted sizes were 31,576 and 28,418 respectively. In that study, the social respect method was applied to obtain the correction factor. As explained in the Materials and Methods section, for each social respect level, the mean number of replies is divided by the mean number of replies in the third category (i.e., medium respect) to adjust the replies. This means that replies in some respect categories might receive a weight greater than 1 and that the adjusted estimate can potentially become smaller than the crude estimate. Therefore, we believe that the social respect method does not necessarily reflect the same concept as the VF.

Expert opinion has only been applied in Iran to estimate visibility for abortion [18]. A second study conducted in Iran that used the game of contacts suggested that the visibility determined based on experts’ opinions was higher than that obtained from the perspective of female who had an abortion [27]. This might be partially justified by the fact that experts usually provide services to hidden groups. Therefore, they are in close contact with these groups and are likely to overestimate how visible the behavior is.

Another method applied in Iran was the comparison of the results obtained using the CW method or PRM with those obtained using NSU. These studies were conducted in an educated population. It is difficult to estimate how successful this approach would be in the general population. There is no evidence of the successful application of a CW study in the Iranian general population.

The coming-out rate is essentially the game of contacts, as it divides the average number of acquaintances by the average network size. In the game of contacts, data from both the numerator and denominator are collected from the hidden group. However, in the Japanese coming-out rate study, the denominator was the average network size of the general population. One drawback of this method is that the network size of members of marginalized populations might be smaller than that of members of the general population. We believe that the visibility of 1.4% in the Japanese study, which led to a correction factor of about 71, is not realistic, as the denominator was the average network size of the general population.

The main drawback of the method is that the crude size is estimated based on replies from members of general population. However, the correction factor is estimated from the perspective of members of the hidden group. We believe that there is room to develop more concrete methods that gather both pieces of information from the same population.

A second limitation of the game of contacts is that access to a random sample of members of the hidden population may not be simple to acquire. For example, in the study of Maghsoudi et al. [19], the authors interviewed 76 FSWs who received services from DICs. This group forms the most vulnerable part of the FSW population, and it is highly likely that they sell sex in exchange for money to defray the costs of their addiction.

One important limitation of the published manuscripts was that not all studies provided a confidence interval (CI). As not enough details were given in the manuscripts, it was not possible to estimate the lower and upper bounds. For example, social respect is calculated by dividing 2 means, but manuscripts simply reported the final statistic. Various approaches can be applied to provide a CI or plausibility range for the VF. In the expert opinion approach, minimum and maximum values can be applied as a plausibility range. For other methods, bootstrapping is an option. This means drawing several samples with replacement from the original sample. Each sample can be analyzed independently to provide the empirical distribution of the VF. Finally, the 2.5 and 97.5th percentiles can be calculated as the lower and upper bounds of the CI.

We also tried to merge the estimates via a meta-analysis to obtain a combined estimate for each behavior. As mentioned above, we were not able to estimate the standard error for all studies. Furthermore, the results were hugely heterogeneous. Issues such as the method of estimation and region might partially explain this heterogeneity. Therefore, we believe that there is still a need to develop concrete methodological frameworks for future studies.

The VF has been used not only to correct the size estimation of stigmatized behaviors, but also to assess the completeness of cancer registries [28]. To do so, 1995 face-to-face sex-matched interviews with members of the general population were conducted. In the first step, NSU was applied to estimate the number of cancer patients. Network size was confined to relatives, with relationships broken down into a comprehensive list. The summation method was applied to estimate the network size. Respondents were asked to count the number of living cancer patients in each relationship. These data were used to provide the crude number of cancer patients. To address visibility, an independent study was organized among 415 cancer patients. The game of contact methodology was followed to estimate the VF for each cancer type. The overall visibility was 86.0% (95% CI, 83.0 to 89.0). The adjusted results were compared with cancer registry statistics. The completeness was calculated as about 66.0%.

Many published NSU analyses did not correct their results to address the visibility issue [6,10,32]. Our review revealed that even in European countries where most of the hidden behaviors are not against the law, the visibility was nonetheless low. This highlights the need to correct for transparency bias in size estimation studies.

We found a manuscript that applied an unusual method of tackling the visibility issue. Kadushin et al. [6] recruited individuals aged 16-44 to estimate the number of heroin users in the US. They estimated the average network size as 55. The reference groups used to estimate the average network size included the number of those who were attacked, hit, or burglarized in the last year. The authors assumed that when the visibility of the reference groups used to estimate the network size and the hidden characteristics are the same, no extra VF factor is needed. One drawback of this method is that the estimated network size is not indeed the network size. In other words, the value of 55 does not reflect the average network size of the general population and cannot be used in independent studies.

Visibility is the most important concern in NSU studies. However, there are other sources of error in NSU studies, such as the barrier effect [33]. In the NSU method, it is assumed that respondents have an equal chance of knowing members of the reference group. However, there might be some reference groups that are more known by respondents in a specific sex or age cohort. This error is known as the barrier effect. In Thailand, a national survey among 3,790 respondents was conducted [10]. The network size was estimated using 19 reference groups. To minimize the barrier effect and transmission error in network size estimation, the method proposed by McCormick et al. [34] was applied. McCormick et al. [34] argued that an uneven distribution of reference groups in the society causes serious problems in estimations of network size. They proposed a latent non-random mixing model to take into account the issue of unevenness. They also proposed a much easier approach based on scaling-down the reference groups. Suppose a male name is selected as a reference group, and it is known that 10.0% of the male population aged 20-30 has that name. Then, 10.0% of the respondents should be males in the same age group. To avoid this problem, we recommend using names with an even distribution across time as reference groups.

In the study conducted in Moldova, the authors proposed an age adjustment but did not apply it in their analysis [8]. They assumed that the age of the respondent may influence the number of PWID in his or her personal network. Therefore, they proposed stratifying the analysis by age categories. The weighted mean of NSU estimates was used as the final estimate, where weights were based on the sample size in each age group.

## CONCLUSION

While NSU is a promising size estimation tool, the VF is not available for most settings, in particular the Middle East and North Africa region, with the exception of Iran. All methods applied to estimate the VF are prone to some biases. More research is needed to develop more concrete methodologies to fill these gaps.

## Figures and Tables

**Table 1.** Summary of methods used to estimate the VF

Method	Samples	Work	Strengths	Weaknesses
Expert opinion	Field experts	Approach field experts and ask their opinion	Easy to apply	Might overestimate the VF
			Needs no contact with the hidden group	
Comparison of NSU with PRM	Hidden and general population	Use ratio of NSU estimates to estimates made using another method	Provides 2 estimates	Difficult to implement
			Helps to validate the estimates	
Social respect	General population	Weighted number of alters known by participant	Needs no contact with the hidden group	Does not reflect the VF concept; adjusted estimate might be smaller than the crude estimates
			Extracts estimates and correction factors from a single population	
Coming-out ratio	Hidden population	Divide total number of aware alters by the total number of alters	NA	Needs contact with the hidden group
				Needs estimation of the network size of the hidden population
				Extracts estimates and correction factors from 2 populations
Game of contacts	Hidden population	Divide total number of aware alters by the total number of alters in a limited network	Applied in most settings and in different countries	Needs contact with the hidden group
				Extracts estimates and correction factors from 2 populations

VF, visibility factor; NSU, network scale-up; PRM, proxy respondent method; NA, not applicable.

**Table 2.** Summary of studies that reported visibility factor

Behavior	Country, year [reference]	Sample (n)	Method	Transparency (95% CI), %
PWID	Ukraine, 2009 [9]^1^	PWIDs (28)	Game of contacts	57.0
	China (Chongqing), 2013 [26]	Participants admitted knowing PWID (113)	Social respect	50.0
	Iran, 2014 [19]	PWIDs (163)	Game of contacts	54.0 (50.0, 58.0)
	Iran, 2014 [25]	Students (420)	Comparison of NSU with PRM	Female: 0.0
				Male: 35.0
				Aggregate: 14.0
	Georgia, 2015 [29]	PWID (1,951)	Game of contacts	Tbilisi: 46.2 (41.0, 51.4)
				Gori: 34.8 (29.3, 40.2)
				Telavi: 32.0 (26.6, 37.4)
				Zugdidi: 46.1 (40.3, 51.9)
				Batumi: 45.4 (39.5, 51.3)
				Kutaisi: 44.4 (38.6, 50.1)
				Rustavi: 34.5 (28.6, 40.4)
				Aggregate: 40.5 (38.3, 42.6)
	Moldova, 2010 [8]	Not provided	Social respect	Stratified by region: 14.0, 5.0, 76.0, 42.0, 3.9, 44.0, 100.0
FSW	Ukraine, 2009 [9]^1^	FSWs (21)	Game of contacts	34.0
	China (Chongqing), 2013 [26]	Participants admitted knowing FSW (229)	Social respect	111.0
	Iran, 2014 [19]	FSWs (76)	Game of contact	45.0 (42.0, 48.0)
Clients of FSW	China (Chongqing), 2013 [26]	Participants admitted knowing clients (480)	Social respect	45.0
MSM	Ukraine, 2009 [9]^1^	MSMs (108)	Game of contacts	24.0
	China (Shanghai), 2015 [20]	Participants admitted knowing MSM (622)	Social respect	52.0
	China (Chongqing), 2013 [26]	Participants admitted knowing MSM (113)	Social respect	44.0
	Georgia, 2016 [14]	MSMs (210)	Game of contacts	26.0 (23.0, 29.0)
	Rwanda, 2011 [11]	MSMs (17)	Game of contacts	20.0
	Japan, 2012 [13]	Internet users (1,500)	Came out ratio	1.4
Abortion	Iran, 2014 [18]	Midwives and gynecologists (34)	Expert opinion	Therapeutic: 43.0, 73.0
				Spontaneous: 20.0, 34.0
				Intentional: 20.0, 34.0
	Iran, 2016 [27]	For each type (74)	Game of contacts	Therapeutic: 60.0 (54.0, 66.0)
				Spontaneous: 50.0 (43.0, 57.0)
				Intentional: 8.0 (6.0, 10.0)
Alcohol	Brazil, 2011 [12]	Users (294)	Game of contacts	<76.0
	Iran, 2016 [21]	Students (563)	Comparison of NSU and CW	48.0
	Iran, 2014 [25]	Students (420)	Comparison of NSU with PRM	Female: 19.0; male: 48.0
Drug	Brazil, 2011 [12]	Drug users (294)	Game of contacts	Cocaine paste: 72.0
				Marijuana: 78.0
				Crack: <77.0
				Amphetamine: <76.0
				Cocaine powder: <75.0
				Ecstasy: <75.0
	Iran, 2016 [21]	Students (563)	Comparison of NSU and CW	Methamphetamine: 17.0
	Iran, 2014 [25]	Students (420)	Comparison of NSU with PRM	Opium: 6.0 for female; 32.0 for male
Cancer	Iran, 2015 [28]	Cancer patients (415)	Game of contacts	86.0 (83.0, 89.0)

CI, confidence interval; PWID, people who inject drugs; MSM, male who have sex with male; FSW, female sex worker; NSU, network scale-up; PRM, proxy respondent method; CW, cross-wise.

1 In Ukraine study two correction factors were applied; Correction factors were calculated using social respect and game of contact approaches; Results of game of contact are provided in Table 1.
